# Structural and functional brain network correlates of depressive symptoms in premanifest Huntington's disease

**DOI:** 10.1002/hbm.23527

**Published:** 2017-03-15

**Authors:** Peter McColgan, Adeel Razi, Sarah Gregory, Kiran K. Seunarine, Alexandra Durr, Raymund A.C. Roos, Blair R. Leavitt, Rachael I. Scahill, Chris A. Clark, Doug R. Langbehn, Geraint Rees, Sarah J. Tabrizi, Track On‐HD Investigators

**Affiliations:** ^1^ Department of Neurodegenerative Disease UCL Institute of Neurology London WC1N 3BG United Kingdom; ^2^ Wellcome Trust Centre for Neuroimaging, UCL Institute of Neurology London WC1N 3BG United Kingdom; ^3^ Department of Electronic Engineering, NED University of Engineering and Technology Karachi Pakistan; ^4^ Developmental Imaging and Biophysics Section UCL Institute of Child Health London WC1N 1EH United Kingdom; ^5^ APHP Department of Genetics, Groupe Hospitalier Pitié‐Salpêtrière, and Institut du Cerveau et de la Moelle, INSERM U1127, CNRS UMR7225, Sorbonne Universités – UPMC Université Paris VI UMR_S1127 Paris France; ^6^ Department of Neurology Leiden University Medical Centre 2300RC Leiden The Netherlands; ^7^ Centre for Molecular Medicine and Therapeutics Department of Medical Genetics, University of British Columbia 950 West 28th Avenue Vancouver British Columbia V5Z 4H4 Canada; ^8^ Department of Biostatistics University of Iowa Iowa City Iowa; ^9^ National Hospital for Neurology and Neurosurgery Queen Square, London WC1N 3BG United Kingdom

**Keywords:** Huntington's disease, brain network, depression, functional MRI, diffusion tractography

## Abstract

Depression is common in premanifest Huntington's disease (preHD) and results in significant morbidity. We sought to examine how variations in structural and functional brain networks relate to depressive symptoms in premanifest HD and healthy controls. Brain networks were constructed using diffusion tractography (70 preHD and 81 controls) and resting state fMRI (92 preHD and 94 controls) data. A sub‐network associated with depression was identified in a data‐driven fashion and network‐based statistics was used to investigate which specific connections correlated with depression scores. A replication analysis was then performed using data from a separate study. Correlations between depressive symptoms with increased functional connectivity and decreased structural connectivity were seen for connections in the default mode network (DMN) and basal ganglia in preHD. This study reveals specific connections in the DMN and basal ganglia that are associated with depressive symptoms in preHD. *Hum Brain Mapp 38:2819–2829, 2017*. © **2017 The Authors Human Brain Mapping Published by Wiley Periodicals, Inc.**

## INTRODUCTION

Huntington's disease is a progressive neurodegenerative disease caused by a dominantly inherited CAG repeat expansion in the huntingtin gene on chromosome 4 [Ross et al., 2014]. It is characterized by cognitive, motor and neuropsychiatric impairment. Depression can precede the onset of motor symptoms by many years [Tabrizi et al., 2009] and has a significant impact on morbidity [Beglinger et al., 2010] with a lifetime prevalence of 20% in the pre‐symptomatic or premanifest stage [Julien et al., 2007]. Neuroimaging studies have identified specific brain variations associated with depression in HD including gray matter volume loss and white matter (WM) microstructural abnormalities in the rostral anterior cingulate [Hobbs et al., 2011; Sprengelmeyer et al., 2014], and abnormal task‐based activations in prefrontal cortex [Gray et al., 2013; Unschuld et al., 2012]. While variations in these regions occur generally in HD [Tabrizi et al., 2009], these studies suggest they are particularly affected in HD patients with depression. Characteristics of depression and apathy in Huntington's disease may overlap [Epping et al., 2013]. Brain regions implicated in depression in people without Huntington's disease have also been associated with apathy [Stanton et al., 2013]. However, others report the presence of a distinct apathy syndrome in HD separate from depression [Levy et al., 1998; Naarding et al., 2009], which begins in the premanifest stage [Martinez‐Horta et al., 2016] and progresses over time [Thompson et al., 2012]. Using data from two large multi‐center cohort studies in HD, Track‐HD [Tabrizi et al., 2009], and Track‐On HD [Kloppel et al., 2015], we directly compared how variations in functional and structural brain networks relate to depressive symptoms in premanifest Huntington's disease (preHD) and healthy controls. Apathy and anxiety were also investigated given their potential overlap with depression.

## METHODS

### Cohort

Track‐On HD (2012) [Kloppel et al., 2015] and Track‐HD (2011) [Tabrizi et al., 2009] cohorts were included in this study. The structure‐function analysis was performed in the Track‐On HD cohort and the structural replication analysis was performed in the Track‐HD cohort. The Track‐On HD fMRI cohort included 186 participants (92 preHD and 94 controls) (see Supporting Information table S1). Baltimore self‐reported apathy data was missing from 3 preHD subjects from the fMRI cohort. The diffusion MRI cohort included 151 (70 preHD and 81 controls) (see Supporting Information table S2). The replication analysis included 96 participants with diffusion MRI data only (50 preHD and 46 controls) (see Supporting Information table S3). Of the participants in Track‐On HD, 31 preHD and 29 controls had previously participated in Track‐HD. Although not significant, depression scores differed between preHD and controls (see Supporting Information tables S1‐S3). See Supporting Information methods for detailed inclusion/exclusion criteria.

Evaluation for psychiatric symptoms was performed on the day of MRI scanning by a neurologist or psychiatrist using the Hospital anxiety and depression score (HADS), the Baltimore apathy and irritability scale, and the Beck's depression inventory‐2 (BDI‐II). See Supporting Information tables S4 and S5 for clinical breakdowns of the BDI‐II and HADS depression (HADS‐D) and anxiety scores (HADS‐A). The self‐reported Baltimore apathy scale was chosen as the apathy measure of interest due to incomplete data for the companion reported Baltimore apathy scale. While we acknowledge the possibility of bias or cognitive deficit in self‐reports, comparison of companion reported and self‐reported apathy scores in Huntington's disease shows high correlation suggesting validity of self‐reported apathy in Huntington's disease [Mason and Barker, 2015].

### MRI Acquisition Track‐on HD

3T MRI data were acquired on two different scanners (Philips Achieva at Leiden and Vancouver and Siemens TIM Trio at London and Paris). Diffusion‐weighted images were acquired with 42 unique gradient directions (*b* = 1,000 sec/mm^2^). Eight images with no diffusion weighting (*b* = 0 sec/mm^2^) and one image with no diffusion weighting (*b* = 0 sec/mm^2^) were acquired from the Siemens and Philips scanners, respectively. Voxel size for Siemens scans was 2 × 2 × 2 mm^3^ and for Phillips 1.96 × 1.96 × 2 mm^3^. For resting state fMRI, 165 whole‐brain volumes were acquired at a repetition time of 3s using a T2*‐weighted echo planar imaging (EPI) sequence. Voxel size for both Siemens and Philips scans was 3.3 x 3.3 x 3.3mm^3^. Please see Supporting Information Methods for detailed acquisition parameters.

### MRI Acquisition Track‐HD

3T MRI data were acquired were acquired on Siemens (London and Paris) and Philips (Leiden) 3T MRI scanners. Diffusion‐weighted images with 42 unique gradient directions (*b* = 1,000 sec/mm^2^) were collected with either seven images (Siemens) with no diffusion weighting or one image with no diffusion weighting (Phillips). Voxel size for Siemens scans was 2 × 2 × 2 mm^3^ and for Phillips 1.96 × 1.96 × 2 mm^3^. Please see Supporting Information Methods for detailed acquisition parameters.

### MRI Data Analysis

#### Structural MRI data

Cortical and sub‐cortical regions of interest (ROIs) were generated by segmenting a T1‐weighted image using the Freesurfer Desikan atlas [Desikan et al., 2006]. The globus pallidus, nucleus accumbens and amygdala were excluded, as automatic segmentation of these regions is not sufficiently reliable [Hibar et al., 2015]. The cerebellum was excluded due partial coverage necessary to maintain acceptable imaging times. This resulted in 70 cortical regions and 6 subcortical regions (caudate, putamen, and thalamus bilaterally).

### Diffusion Tensor Imaging Data

#### Data pre‐processing

For the diffusion data the *b* = 0 image was used to generate a brain mask using FSL's brain extraction tool [Smith, 2002]. Eddy correct was used to align the diffusion‐weighted volumes to the first *b* = 0 image and the gradient directions updated to reflect the changes to the image orientations. Finally, data were reconstructed using DTI and constrained spherical deconvolution, as implemented in MRtrix [Tournier et al., 2012]. Freesufer ROIs were warped into diffusion space by mapping between the T1‐weighted image and fractional anisotropy (FA) map using NiftyReg [Modat et al., 2010] and applying the resulting warp to each of the ROIs. A foreground mask was generated by combining Freesurfer segmentations with the WM mask. A summary of the processing pipeline is provided in Supporting Information Figure S1.

#### Diffusion tractography

Whole brain probabilistic tractography was performed using the iFOD2 algorithm in MRtrix [Tournier et al., 2012]. Specifically, five million streamlines were seeded throughout the WM, in all foreground voxels where FA > 0.2. Streamlines were terminated when they either reached the cortical or subcortical grey‐matter mask or exited the foreground mask. The spherical deconvolution informed filtering of tractograms algorithm [Smith et al., 2013] was used to reduce biases. The resulting set of streamlines was used to construct the structural brain network.

### Functional MRI

#### Data pre‐processing

Preprocessing was performed using SPM8 and the CONN functional connectivity toolbox version 14 (https://www.nitrc.org/projects/conn/) [Whitfield‐Gabrieli and Nieto‐Castanon, 2012] running under MATLAB *v*8.3. Segmented images were used to create an improved anatomical scan for coregistration. The first four EPI images were discarded to allow for steady state equilibrium. Functional images were first realigned, incorporating field maps for inhomogeneity correction whenever available and then coregistered to the new anatomical image. Freesurfer ROIs were also coregistered to the anatomical image using NiftyReg [Modat et al., 2010]. In CONN regression of noise ROIs (without global signal regression) was carried out using the anatomical Compcorr method [Behzadi et al., 2007], along with six movement parameters, followed by band‐pass filtering between 0.009 and 0.08 Hz, calculation of bivariate correlations and application of a Fisher transform. See Supporting Information Methods for more detailed information.

#### Construction of structural and functional connectivity matrices

For structural connectivity matrices ROIs were defined as connected if a fiber originated in ROI 1 and terminated in ROI 2. For functional matrices ROIs were defined as functionally connected if there was a correlation between the time series of ROI 1 and ROI 2. Structural connections were weighted by streamline count, while functional connections were weighted by magnitude of correlation. Connections were then combined into 76 × 76, undirected and weighted matrices. Thresholding was applied to remove weak spurious connections [Rubinov and Sporns, 2010]. For both structural and functional connectivity matrices only those connections present in 75% of controls subjects were retained, consistent with thresholding strategies used in the literature [McColgan et al., 2015; van den Heuvel and Sporns, 2011). Binary matrices were created by converting the weights in matrices to 0 or 1 to denote the absence or presence of a connection. A significant correlation between connectivity and a clinical variable in the context of binary matrices suggests that there is a relationship between the magnitude of the clinical correlation and the likelihood that the connection is present.

#### Cortical modules and depression

A cortical module associated with depression was first identified. This was done by calculating an average functional connectivity matrix across participants, for cortical regions only. This was then decomposed in a data‐driven manner using the Louvain method for community detection (Blondel et al., 2008] as implemented in the Brain connectivity toolbox [Rubinov and Sporns, 2010] (See Supporting Information methods for additional information). Pearson and Spearman rank partial correlations were then performed between depression scores HADS‐D and BDI‐II and the total number of functional connections within each module. Age, gender, site, and CAG repeat length were included as covariates. The brain regions in the module showing correlations with depression were then combined with the caudate and thalamus, as these regions are implicated in depression [Gong and He, 2015] and are selectively vulnerable in preHD [McColgan et al., 2015]. This network was then used an input in the network‐based statistic (NBS) correlation analysis to identify which specific connections within the module are associated with HADS‐D and HADS‐A, BDI‐II and the self‐reported Baltimore apathy scale (see next section).

#### Network‐based statistics

Using this method, a test statistic is calculated for each connection independently. A primary threshold (*P* < 0.05, uncorrected) is then applied to form a set of supratheshold connections. Permutation testing is then used to calculate a family‐wise error (FWE) corrected *P*‐value for each set of suprahreshold connections or sub‐network (please see Supporting Information methods for more details) [Zalesky et al., 2010]. Results reaching FWE corrected *P* < 0.05 are reported as significant, with *P*‐values relating to the significance of all the connections within a sub‐network as a whole as opposed to individual connections. Both binary and weighted networks were investigated as both have been reported in the depression literature [Gong and He, 2015]. Age, gender, site were included as covariates for the between group analysis and control correlation analysis. For the preHD correlation analysis, CAG repeat length was included as an additional covariate. Group analyses between depressed and non‐depressed preHD participants were not performed due to a limited number of preHD participants reaching moderate or severe depression. This was also the case for control groups. Thus, we focused on correlations across the spectrum of depressive symptoms as opposed to clinically significant depression.

Based on observations from the literature that depression shows positive correlation with functional connectivity [Li et al., 2013; Perrin et al., 2012; Sheline et al., 2010] and negative correlation [Korgaonkar et al., 2014] with structural connectivity in the default mode network (DMN) and basal ganglia [Gong and He, 2015], we report positive correlations in the resting state fMRI analysis and negative correlations in the diffusion MRI analysis. Reciprocal correlations (i.e., negative correlation with functional connectivity and positive correlation with structural connectivity) were also tested and are reported in full Supporting Information.

#### Replication analysis

The structural connectivity analysis was replicated in the separate Track‐HD 2011 cohort.

#### Off medication analyses

To account for the effect of antidepressant medication NBS analyses for depression were repeated with inclusion of binary covariate, where 1 denoted those taking antidepressant medication within 30 days of the MRI scan and 0 denoting those not on antidepressant medication during this time period. While we acknowledge pharmacological heterogeneity, the inclusion of a binary covariate allows us to account for common sources of variance in the data associated with medication. While a binary covariate may not fully capture, the subtleties of every type of pharmacological heterogeneity, such heterogeneity is likely to be uncorrelated with apathy and so serve as noise in the analysis, reducing our power to detect effects but not calling into question any of the effects we have actually identified.

## RESULTS

### Within Modular Functional Connectivity and Depression

**Figure 1:**
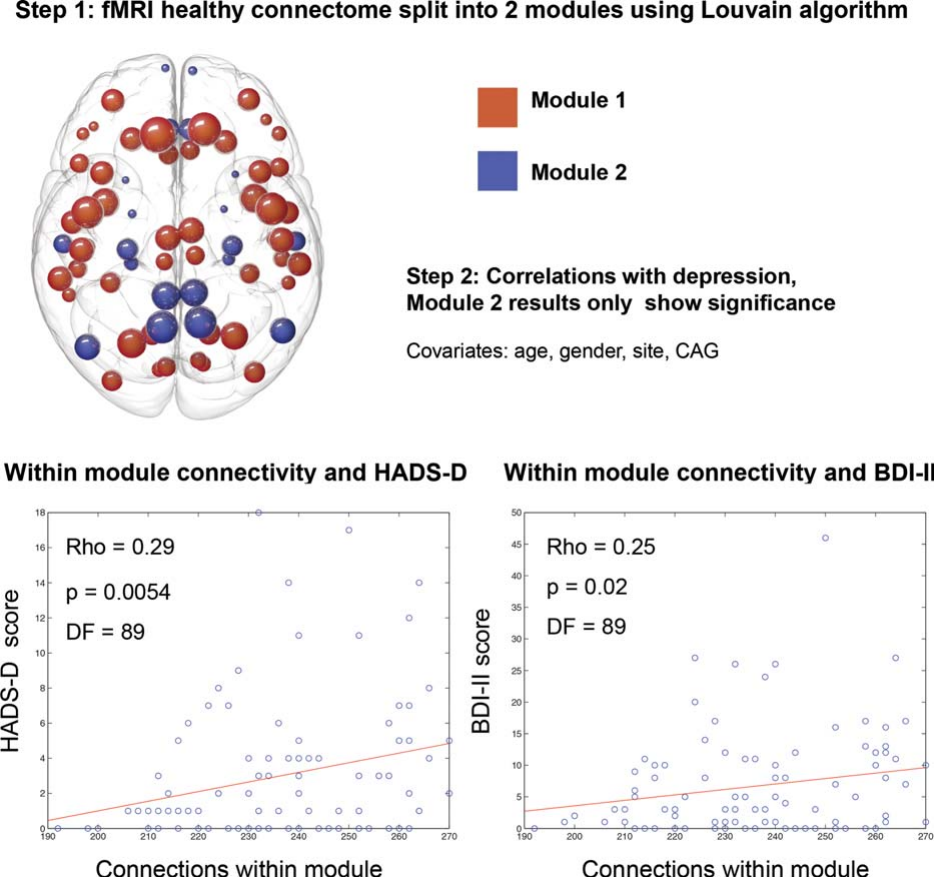
Cortical modules and total within module (2) connectivity correlation with depression scores. Spheres represent brain regions. Red – module 1 and purple – module 2. Significant Pearson partial correlations between total within module functional connectivity and HADS‐D and BDI‐II scores were only seen with module 2. Rho = correlation coefficient, *P* = *P*‐value, DF = degrees of freedom. [Color figure can be viewed at http://wileyonlinelibrary.com]

The average functional cortical matrix was split into two modules, defined in a data driven manner using the Louvain method for community detection, (see Supporting Information for details). Module 1 contained 48 regions, while module 2 contained 22 regions (see Supporting Information table S6). For Pearson partial correlations, the total number of connections within module 2 showed significant correlation with both HADS‐D (*df* = 89, Rho = 0.29, *P* = 0.0054) and approached significance after Bonferroni correction for BDI‐II (*df* = 89. Rho = 0.25, *P* = 0.02; see Fig. 1). Similarly for Spearman rank correlations the total number of connections within module 2 showed significant correlation with both HADS depression (*df* = 89, Rho = 0.29, *P* = 0.0059) and approached significance after Bonferroni correction for BDI‐II (*df* = 89. Rho = 0.22, *P* = 0.051). We observed that all connections in module 2 have been reported previously as belonging to the DMN [Buckner et al., 2008]. No correlations were seen with module 1. Results reported relate to the binarized matrix as no significant correlations were found with the weighted functional matrix.

### Depression Correlates with Increased Functional Connectivity in preHD

**Figure 2:**
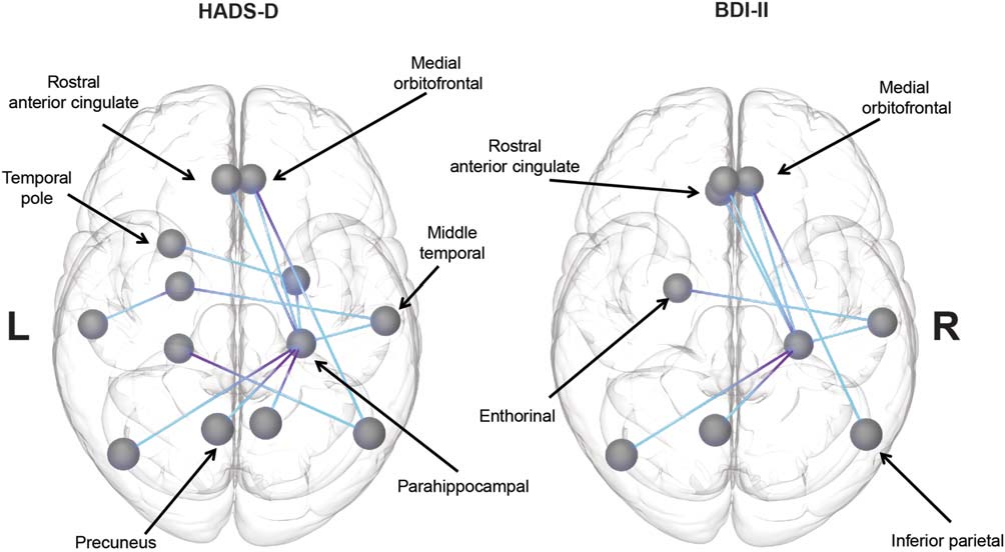
Track‐On HD fMRI cohort: NBS analysis displaying connections that show positive correlation with depression scores for resting state fMRI in preHD. Blue lines indicate significant correlations between functional connections and HADS‐D (*df* = 89, *P* = 0.008) and BDI‐II (*df* = 89, *P* = 0.026) depression scores. [Color figure can be viewed at http://wileyonlinelibrary.com]

Positive correlations were seen between depression score and functional connectivity (binary matrices only) for both HADS‐D (*df* = 89, *p* ^FWE^ = 0.008) and BDI‐II (*df* = 89, *p* ^FWE^ = 0.026), notably in the connections between the rostral anterior cingulate, medial orbitofrontal, precuneus and parahippocampal regions. No significant correlations were seen with depression score and functional connectivity for the controls or for weighted networks. See Figure 2 and Supporting Information table S7 and summary Supporting Information table S14. Additionally no significant reciprocal (negative correlations) were observed. See Supporting Information table S15.

### Depression Correlates with Reduced Structural Connectivity in preHD

**Figure 3:**
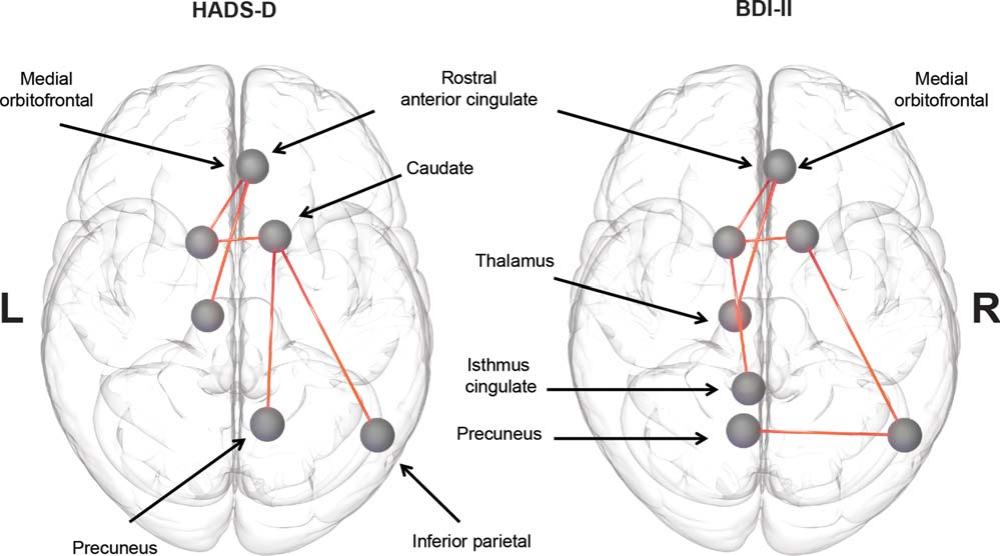
Track‐On HD diffusion MRI cohort: NBS analysis displaying connections that show negative correlation with depression scores for diffusion MRI in preHD. Red lines indicate significant positive correlations between structural connections and HADS‐D (*df* = 67, *P* = 0.036) and BDI‐II (*df* = 67, *P* = 0.019) depression scores. [Color figure can be viewed at http://wileyonlinelibrary.com]

Negative correlations were seen between depression score and structural connectivity (binary matrices only) for both HADS‐D (*df* = 67, *p* ^FWE^ = 0.036) and BDI‐II (*df* = 67, *p* ^FWE^ = 0.019), notably in the connections between the rostral anterior cingulate, medial orbitofrontal, precuneus and caudate and thalamus regions. No significant correlations were seen with depression score and structural connectivity for the controls. See Figure 3 and Supporting Information table S8 and summary Supporting Information table S14. Additionally no significant positive correlations were observed. See Supporting Information table S15.

### Apathy Correlates with Increased Functional but Not Structural Connectivity in preHD

**Figure 4:**
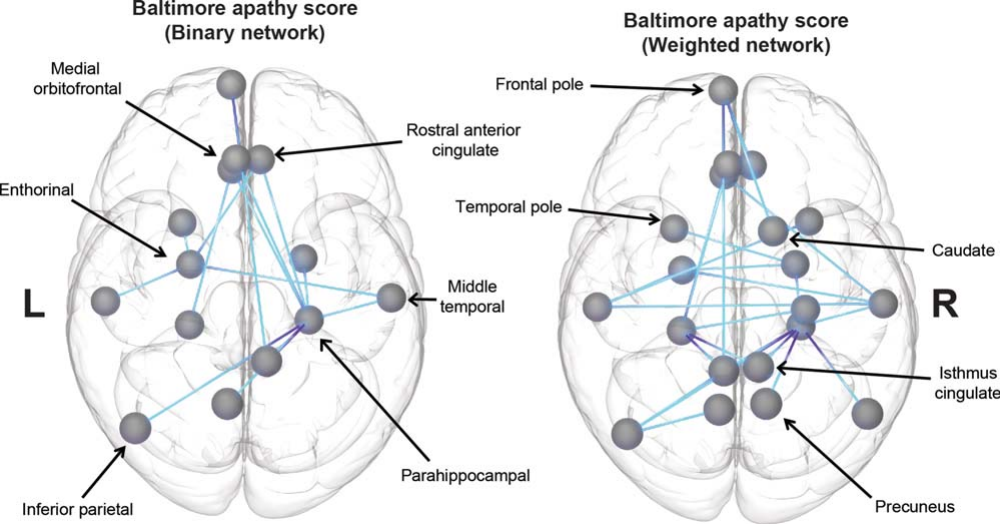
Track‐On HD fMRI cohort: NBS analysis displaying connections that show positive correlation with Baltimore self‐reported apathy score for resting state fMRI in preHD. Blue lines indicate significant negative correlations between binary (*df* = 86, *P* = 0.036) and weighted (*df* = 86, *P* = 0.005) functional connections and Baltimore self‐reported apathy score. [Color figure can be viewed at http://wileyonlinelibrary.com]

Positive correlations were seen between self‐reported apathy and both binary (*df* = 86, *p* ^FWE^ = 0.005) and weighted functional matrices (*df* = 86, *p* ^FWE^ = 0.034); however, no correlation with apathy and structural (binary or weighted) matrices was observed. See Figure 4 and Supporting Information tables S9 and S16. HADS‐A showed no correlation with functional or structural (binary or weighted) connectivity matrices (See Supporting Information table S17). This suggests that the correlations we demonstrate between depression scores and connectivity are specific for depressive symptoms and not anxiety.

### Structural Connectivity Replication Analysis

**Figure 5:**
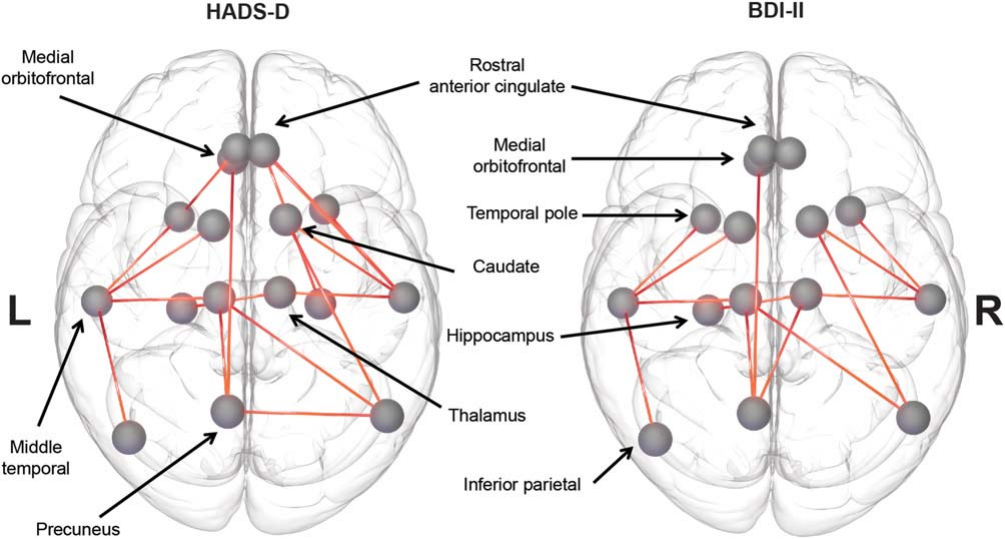
Track‐HD diffusion MRI replication cohort. NBS analysis displaying connections that show negative correlation with depression scores for diffusion MRI in preHD. Red lines indicate significant positive correlations between structural connections and HADS‐D (*df* = 67, *P* = 0.036) and BDI‐II (*df* = 67, *P* = 0.019) depression scores. [Color figure can be viewed at http://wileyonlinelibrary.com]

For weighted connectivity matrices, negative correlations were seen between depression score and structural connectivity for both HADS‐D (*df* = 47, *p* ^FWE^ = 0.014) and BDI‐II (*df* = 47, *p* ^FWE^ = 0.045), notably in the connections between the rostral anterior cingulate, medial orbitofrontal, precuneus and caudate and thalamus regions (See Fig. 5). No significant correlations were seen between depression score and structural connectivity for the controls. No significant correlations were seen between binary matrices and depression scores for either group. See Supporting Information table S10 and summary Supporting Information table S14. Additionally no significant reciprocal (positive) correlations were observed. See Supporting Information table S15.

### Group Differences between preHD and Controls

**Figure 6:**
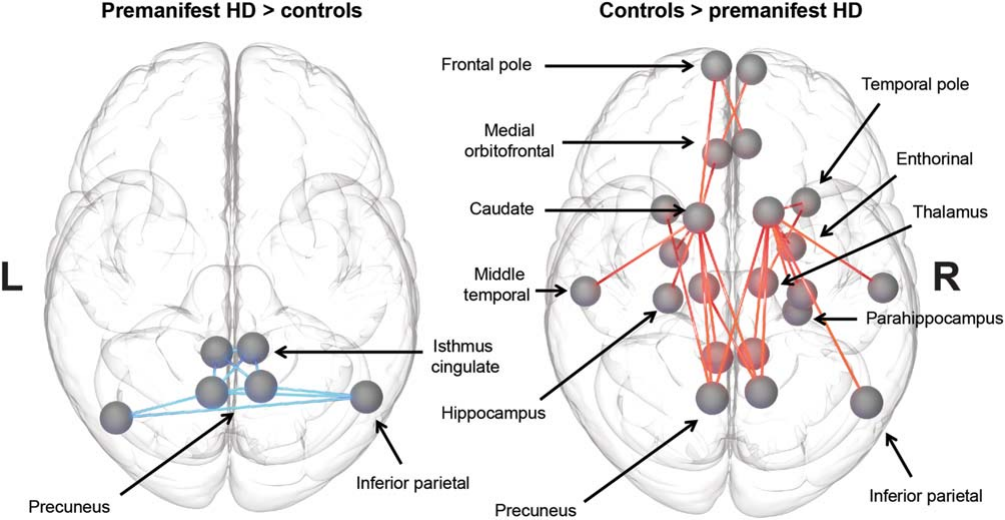
NBS analysis displaying connections that show group differences in preHD versus controls for resting state fMRI (Track‐On HD) and diffusion MRI (Track‐HD) cohorts. Blues lines indicate significance for preHD > controls (*P* = 0.036), while red lines indicate significant connections for controls > preHD (*P* = 0.0144). [Color figure can be viewed at http://wileyonlinelibrary.com]

For cohorts with no significant group differences in depression scores (Track‐On HD fMRI and Track‐HD diffusion), preHD participants showed greater functional connectivity (binary matrices only) between precuneus, isthmus cingulate and inferior parietal regions bilaterally compared to controls (*p* ^FWE^ = 0.036) and reduced structural connectivity between basal ganglia hubs and cortical regions including the precuneus, isthmus cingulate, inferior parietal, and medial orbitofrontal compared to controls (*p* ^FWE^ = 0.018), (weighted matrices only). See Figure 6 and Supporting Information tables S11 and S12. Similarly for the Track‐On HD diffusion cohort reduced structural connectivity was seen between basal ganglia hubs and cortical regions (*p* ^FWE^ = 0.035) (weighted matrices only); however, in this cohort there were significant group differences in depression scores raising the possibility that depression may be driving this result. See Supporting Information table S13.

### Off Medication Analyses

All analyses in the study were repeated with the use of anti‐depressant medications included as a covariate and revealed similar results. A binary covariate was used with 1 denoting use of antidepressants within 30 days prior to MRI scanning and 0 denoting no antidepressant use within this time period. The Track‐On HD fMRI cohort had 33 preHD and 11 controls on anti‐depressants, the diffusion tractography cohort had 25 preHD and 10 controls on anti‐depressants and the Track‐HD replication cohort had 8 preHD and 3 controls on anti‐depressants. See summary Supporting Information table S14.

## DISCUSSION

In this study, brain network connections that correlated with depressive symptoms and apathy scores in preHD were identified. Positive correlations between depressive symptoms and apathy scores were seen with functional connections, predominantly between the default mode regions, while negative correlations in structural connections were seen between the cortex and basal ganglia (for depressive symptoms but not apathy). Furthermore these connectivity variations associated with depressive symptoms were also seen in similar regions when comparing preHD and control groups, when depression scores were not significantly different between groups.

Previous neuroimaging studies have identified brain regions associated with depression in HD. In preHD and manifest HD, there is a negative correlation between grey matter volume loss of the rostral anterior cingulate and BDI score [Hobbs et al., 2011]. WM microstructural abnormalities have also been identified in early HD and preHD patients with depressive symptoms using diffusion tensor imaging (DTI). Variations in FA are seen in anterior cingulate, ventromedial frontal cortex, superior frontal cortex, insula and cerebellum [Sprengelmeyer et al., 2014]. Similarly task‐based fMRI reveal an association between depression and activation of the ventromedial prefrontal cortex in preHD [Unschuld et al., 2012] and activation of dorsolateral prefrontal cortex in manifest HD [Gray et al., 2013]. Similar findings were observed in the present study with respect to both structural and functional connectivity of the rostral anterior cingulate and medial orbitofrontal regions. Loss of structural connectivity between medial orbitofrontal and thalamus, hippocampus and frontal pole are also seen between preHD and controls.

Our results also show marked similarities to connectivity variations in major depressive disorder without HD. We demonstrate in preHD that depression scores are positively correlated with the functional connectivity of a brain module which includes the precuneus, isthmus cingulate, inferior parietal, hippocampus, parahippocampal gyrus, entorhinal, temporal pole, rostral anterior cingulate, and medial orbitofrontal. These regions are found in the default mode brain network [Buckner et al., 2008], which shows increases in functional connectivity in those with MDD [Greicius et al., 2007]. The DMN can be further sub‐divided into anterior and posterior components. The anterior DMN consists of the medial prefrontal, posterior cingulate and parietal regions, while the posterior DMN includes the posterior cingulate and parietal regions [Damoiseaux et al., 2008; Li et al., 2013]. Using independent components analysis of resting state fMRI data [Li et al., 2013] demonstrated increased functional connectivity in anterior and posterior default mode sub‐networks in MDD. In keeping with this, using a brain network approach, we demonstrate similar findings in preHD participants, showing a correlation between depressive symptoms and increased functional connectivity between anterior and posterior regions of the DMN.

With respect to the structural networks, a recent study in MDD revealed reduced structural connections between two sub‐networks, one including the precuneus, isthmus cingulate and rostral anterior cingulate and the other including the thalamus, caudate and medial orbitofrontal regions [Korgaonkar et al., 2014]. In keeping with this, we found loss of structural connectivity between medial orbitofrontal, thalamus, caudate, rostral anterior cingulate, precuneus and inferior parietal.

Apathy scores showed positive correlations with functional connections; however no significant correlations were identified between apathy scores and structural connections. The lack of significant correlation with apathy and structural connections suggests apathy may be driven by a functional process, such as neurochemical disturbance as opposed to underlying structural variation. Indeed dopamine modulation is thought to play a role in apathy related to Parkinson's disease thus a similar mechanism may account for apathy in HD [Sinha et al., 2013]. Functional connections correlating with apathy, including medial orbitofrontal and cingulate connections to the parahippocampal gyrus, are consistent with the brain regions implicated in apathy in other neurodegenerative disorders [Benoit et al., 2002; Thobois et al., 2010].

Group differences were found in connections associated with depression comparing preHD and controls, such as functional connections between precuneus, inferior parietal and isthmus cingulate and structural connections between the precuneus, isthmus cingulate, caudate, thalamus and medial orbitofrontal regions. This analysis was performed in cohorts were depression scores did not differ significantly between preHD and controls suggesting that the group differences identified may relate to HD pathology rather the depression.

A small proportion of both our preHD and control participants were taking regular antidepressants. We control for the effect of this by repeating all analyses and including the use of anti‐depressants as a covariate, which provided similar findings to those demonstrated in the main analysis.

While it is difficult to perform large imaging studies in preHD looking specifically at depression and apathy, by conducting a correlation analysis over a range of clinical scores this enabled us to perform the largest imaging study to date looking specifically at depressive symptoms and apathy in HD. One limitation of this approach is how specific these network variations are for preHD related depression rather than depression in general. We try and account for this by showing an absence of correlation between depressive symptoms in controls and functional and structural connectivity, particularly in a control cohort with no significant differences in depression scores relative to preHD. While a group analysis between depressed preHD subjects and non‐depressed preHD subjects may have been preferable, low numbers of those with moderate or severe depression made this unfeasible thus a correlation analysis was performed to examine connectivity relationships over a wide range of depressive symptoms.

The structural and functional Track‐On HD analyses only showed significant results with binary matrices while the replication structural analysis only showed significant results with weighted matrices. Binary matrices indicate the absence or presence of a connection while weighted matrices indicate the strength of a connection. Thus, this discrepancy between cohorts may be due to the fact that the higher depression scores seen in Track‐On HD relate to connection loss, while lower depression scores seen in Track‐HD relate to reductions in connection in strength.

Both the nucleus accumbens and globus pallidus have been implicated in the pathophysiology of apathy [Sinha et al., 2013]. However, we were unable to include these structures in our analysis as automatic segmentation of these regions are not sufficiently reliable [Hibar et al., 2015].

## CONCLUSION

Increased functional connections between the DMN are associated with depressive and apathy symptoms in preHD, while reduced structural connections between the basal ganglia and the DMN are associated with depressive symptoms but not apathy. Furthermore, these connectivity variations associated with depressive symptoms were also present between preHD and control groups, regardless of depression or apathy. These findings reveal the specific functional and structural brain connections implicated in the common neuropsychiatric symptoms occurring in preHD.

## TRACK‐ON HD INVESTIGATORS

B Leavitt, A Coleman, J Decolongon, A. Sturrock, T. Petkau, (University of British Columbia, Vancouver); A Durr, C Jauffret, D Justo, S Lehericy, K Nigaud, R Valabrègue (ICM and APHP, Pitié‐ Salpêtrière University Hospital, Paris). R Roos, A Schoonderbeek, E P ′t Hart (Leiden University Medical Centre, Leiden); H Crawford, E Johnson, M Papoutsi, C Berna, R I Scahill (University College London, London)

## Supporting information

Supporting Information
