# Supplementary material for: Structural and functional brain network correlates of depressive symptoms in premanifest Huntington's disease
Source: Hum Brain Mapp. 2017 Mar 15;38(6):2819–29. doi: 10.1002/hbm.23527 (PMC5434856; doi:10.1002/hbm.23527)
Supplement: Supplementary file 1 — Supporting Information [file HBM-38-2819-s001.docx]

**Supplementary Information**

**Supplementary figures**

**Figure S1. Resting state fMRI and diffusion tractography processing pipelines. Related to ‘MRI data analysis’ section of experimental procedures.** BET = brain extraction tool; CONN = functional connectivity toolbox; CSD = constrained spherical deconvolution; DTI = diffusion tensor imaging; FA = fractional anisotropy; fODF = fibre orientation distribution function; GM = grey matter; QC = quality control, WM = white matter; SPM = statistical parametric mapping.

**
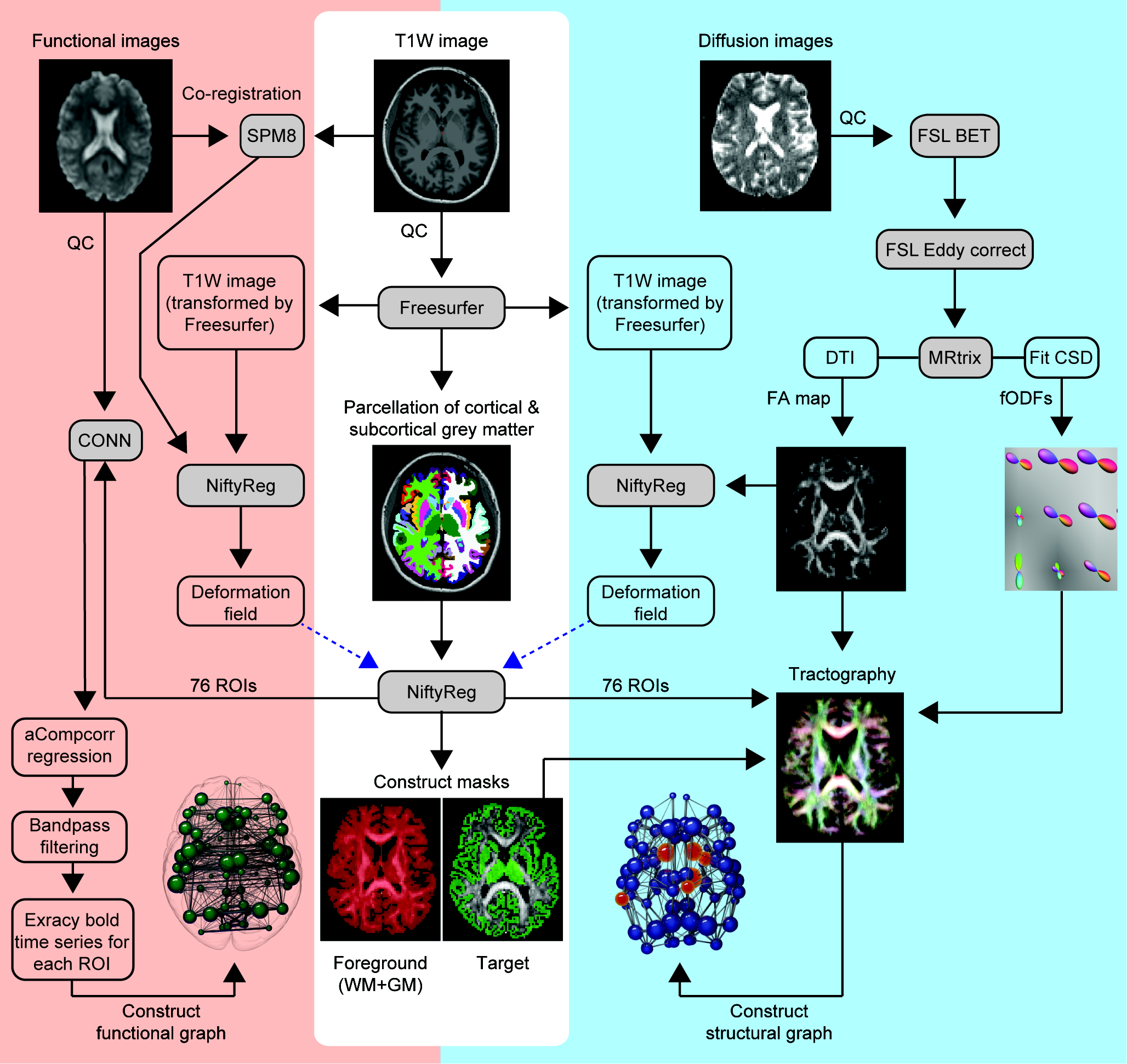
**

**Supplemental tables**

**Table S1. Related to ‘Cohort’ section of methods. Track-On HD fMRI cohort.** SD = standard deviation, M = male, F = female, N = number. ISCED = International standard classification of education. CAG = CAG repeat expansion length, DBS = disease burden scale (Penney, et al., 1997)

|  | **Premanifest HD** | **Control** | **Statistical test** | **P-value** |
| --- | --- | --- | --- | --- |
| **N** | 92 | 94 | - | - |
| **Age (SD)** | 41.8 (9.5) | 47.7 (10.6) | 2 tail t-test | 0.0001 |
| **Gender (M/F)** | 49/43 | 38/56 | Chi-square | 0.079 |
| **ISCED (2/3/4/5/6)** | 7/22/27/35/1 | 9/16/32/35/2 | Chi-square | 0.748 |
| **Study Site (N) (Leiden/London/Paris/Vancouver)** | 17/26/24/25 | 24/25/24/21 | Chi-square | 0.673 |
| **CAG (SD)** | 43.1 (2.3) | - | - | - |
| **DBS (SD)** | 301.4 (52) | - | - | - |
| **BDI (SD)** | 6.8 (8.2) | 4.8 (6.1) | 2 tail t-test | 0.071 |
| **HADS-D (SD)** | 3 (4) | 2.1 (2.6) | 2 tail t-test | 0.068 |
| **HADS-A (SD)** | 5 (3.8) | 4.2 (3.3) | 2 tail t-test | 0.126 |
| **BAIS self reported apathy (SD)** | 11.4 (7) | 8.8 (4.8) | 2 tail t-test | 0.004 |

**Table S2. Related to ‘Cohort’ section of methods. Track-On HD Diffusion fMRI cohort.** SD = standard deviation, M = male, F = female, N = number. ISCED = International standard classification of education. CAG = CAG repeat expansion length, DBS = disease burden scale (Penney, et al., 1997)

|  | **Premanifest HD** | **Control** | **Statistical test** | **P-value** |
| --- | --- | --- | --- | --- |
| **N** | 70 | 81 | - | - |
| **Age (SD)** | 42.6 (9.3) | 48.3 (9.8) | 2 tail t-test | 0.0004 |
| **Gender (M/F)** | 38/32 | 31/50 | Chi-square | 0.049 |
| **ISCED (2/3/4/5/6)** | 5/14/23/27/1 | 7/14/28/30/2 | Chi-square | 0.972 |
| **Study Site (N) (Leiden/London/Paris/Vancouver)** | 12/25/18/15 | 18/25/22/16 | Chi-square | 0.841 |
| **CAG (SD)** | 43 (2.3) | - | - | - |
| **DBS (SD)** | 301 (51.9) | - | - | - |
| **BDI (SD)** | 6.9 (8.8) | 4.7 (6.1) | 2 tail t-test | 0.0674 |
| **HADS-D (SD)** | 3 (4.3) | 1.9 (2.6) | 2 tail t-test | 0.043 |
| **HADS-A (SD)** | 5 (3.8) | 4.1 (3.4) | 2 tail t-test | 0.1 |
| **BAIS self reported apathy (SD)** | 11.3 (7.3) | 8.3 (4.1) | 2 tail t-test | 0.0017 |

**Table S3. Related to ‘Cohort’ section of methods. Track-HD Diffusion MRI cohort.** SD = standard deviation, M = male, F = female, N = number. ISCED = International standard classification of education. CAG = CAG repeat expansion length, DBS = disease burden scale (Penney, et al., 1997)

|  | **Premanifest HD** | **Control** | **Statistical test** | **P-value** |
| --- | --- | --- | --- | --- |
| **N** | 50 | 46 | - | - |
| **Age (SD)** | 42.2 (8.9) | 47.7 (9) | 2 tail t-test | 0.004 |
| **Gender (M/F)** | 26/24 | 15/31 | Chi-square | 0.055 |
| **ISCED (2/3/4/5/6)** | 2/7/19/22/0 | 7/9/12/17/1 | Chi-square | 0.192 |
| **Study Site (N) (London/Leiden/Paris)** | 13/21/16 | 11/20/15 | Chi-square | 0.972 |
| **CAG (SD)** | 43 (2.1) | - | - | - |
| **DBS (SD)** | 301.3 (52.5) | - | - | - |
| **BDI (SD)** | 6.1 (7.5) | 5.8 (6.7) | 2 tail t-test | 0.8307 |
| **HADS-D (SD)** | 2.8 (3.5) | 2.5 (2.7) | 2 tail t-test | 0.6405 |
| **HADS-A (SD)** | 4.8 (3.4) | 4.7 (3.6) | 2 tail t-test | 0.909 |
| **BAIS self reported apathy (SD)** | 10.9 (6.3) | 9.4 (3.5) | 2 tail t-test | 0.156 |

**Table S4. Related to ‘Cohort’ section of methods. BDI-II scores**

| **Track-On HD fMRI** | **BDI-II** |  |  |
| --- | --- | --- | --- |
|  |  | **Premanifest HD** | **Controls** |
| 0-13 | Minimal | 79 | 86 |
| 14-19 | Mild | 6 | 3 |
| 20-28 | Moderate | 6 | 4 |
| 29-63 | Severe | 1 | 1 |
|  |  |  |  |
| **Track-On HD Diffusion** | **BDI-II** |  |  |
|  |  | **Premanifest HD** | **Controls** |
| 0-13 | Minimal | 60 | 76 |
| 14-19 | Mild | 3 | 3 |
| 20-28 | Moderate | 6 | 2 |
| 29-63 | Severe | 1 | 1 |
|  |  |  |  |
| **Track-HD Diffusion** | **BDI-II** |  |  |
|  |  | **Premanifest HD** | **Controls** |
| 0-13 | Minimal | 42 | 40 |
| 14-19 | Mild | 3 | 5 |
| 20-28 | Moderate | 4 | 0 |
| 29-63 | Severe | 1 | 1 |

**Table S5. Related to ‘Cohort’ section of methods. HADS-D and HADS-A scores.**

| **Track-On HD fMRI** | **HADS-D** |  |  | **Track-On HD fMRI** | **HADS-A** |  |  |
| --- | --- | --- | --- | --- | --- | --- | --- |
|  |  | **PreHD** | **Controls** |  |  | **PreHD** | **Controls** |
| 0-7 | Normal | 82 | 88 | 0-7 | Normal | 71 | 77 |
| 8 --10 | Borderline | 3 | 5 | 8 --10 | Borderline | 11 | 11 |
| 11 --21 | Abnormal | 7 | 1 | 11 --21 | Abnormal | 10 | 6 |
|  |  |  |  |  |  |  |  |
|  |  |  |  |  |  |  |  |
| **Track-On HD Diffusion** | **HADS-D** |  |  | **Track-On HD Diffusion** | **HADS-A** |  |  |
|  |  | **PreHD** | **Controls** |  |  | **PreHD** | **Controls** |
| 0-7 | Normal | 61 | 76 | 0-7 | Normal | 55 | 67 |
| 8 --10 | Borderline | 3 | 4 | 8 --10 | Borderline | 7 | 9 |
| 11 --21 | Abnormal | 6 | 1 | 11 --21 | Abnormal | 8 | 5 |
|  |  |  |  |  |  |  |  |
|  |  |  |  |  |  |  |  |
| **Track-HD Diffusion** | **HADS-D** |  |  | **Track-HD Diffusion** | **HADS-A** |  |  |
|  |  | **PreHD** | **Controls** |  |  | **PreHD** | **Controls** |
| 0-7 | Normal | 47 | 43 | 0-7 | Normal | 38 | 36 |
| 8 --10 | Borderline | 2 | 2 | 8 --10 | Borderline | 9 | 6 |
| 11 --21 | Abnormal | 1 | 1 | 11 --21 | Abnormal | 3 | 4 |
|  |  |  |  |  |  |  |  |

**Table S6. Related to figure 1. Cortical modules generated using Louvain algorithm**

| **Module 1** |  | **Module 2** |
| --- | --- | --- |
| L.bankssts | R.caudalmiddlefrontal | L.entorhinal |
| L.caudalanteriorcingulate | R.cuneus | L.inferiorparietal |
| L.caudalmiddlefrontal | R.fusiform | L.isthmuscingulate |
| L.cuneus | R.inferiortemporal | L.medialorbitofrontal |
| L.fusiform | R.lateraloccipital | L.middletemporal |
| L.inferiortemporal | R.lateralorbitofrontal | L.parahippocampal |
| L.lateraloccipital | R.lingual | L.precuneus |
| L.lateralorbitofrontal | R.paracentral | L.rostralanteriorcingulate |
| L.lingual | R.parsopercularis | L.frontalpole |
| L.paracentral | R.parsorbitalis | L.temporalpole |
| L.parsopercularis | R.parstriangularis | L.hippocampus |
| L.parsorbitalis | R.pericalcarine | R.hippocampus |
| L.parstriangularis | R.postcentral | R.entorhinal |
| L.pericalcarine | R.posteriorcingulate | R.inferiorparietal |
| L.postcentral | R.precentral | R.isthmuscingulate |
| L.posteriorcingulate | R.rostralmiddlefrontal | R.medialorbitofrontal |
| L.precentral | R.superiorfrontal | R.middletemporal |
| L.rostralmiddlefrontal | R.superiorparietal | R.parahippocampal |
| L.superiorfrontal | R.superiortemporal | R.precuneus |
| L.superiorparietal | R.supramarginal | R.rostralanteriorcingulate |
| L.superiortemporal | R.transversetemporal | R.frontalpole |
| L.supramarginal | R.insula | R.temporalpole |
| L.transversetemporal | R.bankssts |  |
| L.insula | R.caudalanteriorcingulate |  |

**Table S7.** **Related to figure 2.** **Resting state fMRI Track-On HD and depression**: Association between increase in functional connections and HADS (p = 0.008) and BDI (p = 0.026) scores in premanifest HD. ROI – regions of interest, T-stat – t-statistics.

| **Resting state fMRI Track-On HD** |  |  |  |
| --- | --- | --- | --- |
| **HADS-D** | **ROI 1** | **ROI 2** | **T-stat** |
|  | L.entorhinal | L.middletemporal | 2.11 |
|  | L.temporalpole | R.entorhinal | 1.9 |
|  | L.parahippocampal | R.inferiorparietal | 1.71 |
|  | R.inferiorparietal | R.medialorbitofrontal | 1.97 |
|  | L.entorhinal | R.middletemporal | 1.71 |
|  | L.inferiorparietal | R.parahippocampal | 2.28 |
|  | L.precuneus | R.parahippocampal | 2.57 |
|  | L.rostralanteriorcingulate | R.parahippocampal | 2.56 |
|  | R.entorhinal | R.parahippocampal | 1.82 |
|  | R.medialorbitofrontal | R.parahippocampal | 1.91 |
|  | R.middletemporal | R.parahippocampal | 2.74 |
|  | R.parahippocampal | R.precuneus | 1.83 |
|  |  |  |  |
| **BDI-II** | **ROI 1** | **ROI 2** | **T-stat** |
|  | R.inferiorparietal | R.medialorbitofrontal | 1.77 |
|  | L.entorhinal | R.middletemporal | 1.81 |
|  | L.inferiorparietal | R.parahippocampal | 2.66 |
|  | L.medialorbitofrontal | R.parahippocampal | 1.89 |
|  | L.precuneus | R.parahippocampal | 1.99 |
|  | L.rostralanteriorcingulate | R.parahippocampal | 2.33 |
|  | R.medialorbitofrontal | R.parahippocampal | 2.16 |
|  | R.middletemporal | R.parahippocampal | 2.59 |

**Table S8. Related to figure 3.** **Diffusion MRI Track-On HD and depression**: Association between decrease in structural connections and HADS (p = 0.036) and BDI (p = 0.019) scores in premanifest HD. ROI – regions of interest, T-stat – t-statistics.

| **Diffusion MRI Track-On HD** |  |  |  |
| --- | --- | --- | --- |
| **HADS-D** | **ROI 1** | **ROI 2** | **T-stat** |
|  | L.caudate | R.caudate | 2.22 |
|  | R.caudate | R.inferiorparietal | 2.48 |
|  | L.thalamus | R.medialorbitofrontal | 2.4 |
|  | L.caudate | R.medialorbitofrontal | 3.43 |
|  | R.caudate | R.precuneus | 1.73 |
|  | L.thalamus | R.rostralanteriorcingulate | 2.01 |
|  |  |  |  |
| **BDI-II** | **ROI 1** | **ROI 2** | **T-stat** |
|  | L.isthmuscingulate | L.caudate | 1.78 |
|  | L.caudate | R.caudate | 1.73 |
|  | L.precuneus | R.inferiorparietal | 2.03 |
|  | R.caudate | R.inferiorparietal | 2.7 |
|  | L.thalamus | R.medialorbitofrontal | 2.92 |
|  | L.caudate | R.medialorbitofrontal | 3.27 |
|  | L.thalamus | R.rostralanteriorcingulate | 1.83 |

**Table S9. Related to figure 4. Resting state fMRI TrackOn-HD and apathy**: Association between increase in functional connections and Baltimore self-reported apathy in premanifest HD for binary (p = 0.005) and weighted (p = 0.034) networks. ROI – regions of interest, T-stat – t-statistics.

| **Resting state fMRI Track-On HD** |  |  |  |
| --- | --- | --- | --- |
| **Apathy (binary network)** | **ROI 1** | **ROI 2** | **T-stat** |
|  | L.entorhinal | L.middletemporal | 2.91 |
|  | L.medialorbitofrontal | L.parahippocampal | 1.8 |
|  | L.entorhinal | L.temporalpole | 1.76 |
|  | L.frontalpole | R.isthmuscingulate | 1.9 |
|  | L.entorhinal | R.medialorbitofrontal | 2.58 |
|  | L.entorhinal | R.middletemporal | 2.62 |
|  | L.inferiorparietal | R.parahippocampal | 2.28 |
|  | L.medialorbitofrontal | R.parahippocampal | 2.75 |
|  | L.precuneus | R.parahippocampal | 2.22 |
|  | L.rostralanteriorcingulate | R.parahippocampal | 1.88 |
|  | R.entorhinal | R.parahippocampal | 1.95 |
|  | R.isthmuscingulate | R.parahippocampal | 1.93 |
|  | R.medialorbitofrontal | R.parahippocampal | 1.94 |
|  | R.middletemporal | R.parahippocampal | 2.59 |
| **Apathy (weighted network)** | **ROI 1** | **ROI 2** | **T-stat** |
|  | L.inferiorparietal | L.isthmuscingulate | 1.94 |
|  | L.entorhinal | L.middletemporal | 2.37 |
|  | L.isthmuscingulate | L.parahippocampal | 2.57 |
|  | L.medialorbitofrontal | L.parahippocampal | 2.45 |
|  | L.inferiorparietal | L.precuneus | 2.38 |
|  | L.parahippocampal | L.rostralanteriorcingulate | 1.82 |
|  | L.isthmuscingulate | L.frontalpole | 1.97 |
|  | L.medialorbitofrontal | L.frontalpole | 2.02 |
|  | L.frontalpole | R.caudate | 2 |
|  | L.parahippocampal | R.hippocampus | 1.79 |
|  | L.entorhinal | R.entorhinal | 1.78 |
|  | L.temporalpole | R.entorhinal | 2.3 |
|  | L.parahippocampal | R.isthmuscingulate | 2.57 |
|  | L.frontalpole | R.medialorbitofrontal | 2.14 |
|  | L.entorhinal | R.middletemporal | 2.15 |
|  | L.medialorbitofrontal | R.middletemporal | 2.2 |
|  | L.middletemporal | R.middletemporal | 1.85 |
|  | L.inferiorparietal | R.parahippocampal | 2.43 |
|  | L.isthmuscingulate | R.parahippocampal | 1.85 |
|  | R.entorhinal | R.parahippocampal | 2.44 |
|  | R.inferiorparietal | R.parahippocampal | 1.95 |
|  | R.isthmuscingulate | R.parahippocampal | 1.78 |
|  | R.middletemporal | R.parahippocampal | 1.86 |
|  | R.parahippocampal | R.precuneus | 1.72 |
|  | L.middletemporal | R.temporalpole | 1.73 |

**Table S10. Related to figure 5.** **Diffusion MRI Track-HD and depression replication analysis**: Association between decrease in structural connections and HADS (p = 0.014) and BDI (p = 0.045) scores in premanifest HD. ROI – regions of interest, T-stat – t-statistics.

| **Diffusion MRI Track-HD** |  |  |  |
| --- | --- | --- | --- |
| **HADS-D** | **ROI 1** | **ROI 2** | **T-stat** |
|  | L.inferiorparietal | L.middletemporal | 1.74 |
|  | L.medialorbitofrontal | L.precuneus | 2.4 |
|  | L.middletemporal | L.temporalpole | 1.85 |
|  | L.rostralanteriorcingulate | L.temporalpole | 2.22 |
|  | L.middletemporal | L.thalamus | 1.74 |
|  | L.precuneus | L.thalamus | 2.67 |
|  | L.middletemporal | L.caudate | 1.76 |
|  | L.hippocampus | R.thalamus | 2.17 |
|  | L.precuneus | R.inferiorparietal | 2.06 |
|  | L.thalamus | R.inferiorparietal | 2.56 |
|  | R.caudate | R.inferiorparietal | 1.97 |
|  | R.hippocampus | R.medialorbitofrontal | 1.86 |
|  | R.thalamus | R.middletemporal | 3.17 |
|  | R.caudate | R.middletemporal | 3.05 |
|  | R.medialorbitofrontal | R.middletemporal | 1.83 |
|  | L.medialorbitofrontal | R.rostralanteriorcingulate | 1.93 |
|  | L.rostralanteriorcingulate | R.rostralanteriorcingulate | 2.05 |
|  | R.hippocampus | R.rostralanteriorcingulate | 2.29 |
|  | R.middletemporal | R.rostralanteriorcingulate | 1.82 |
|  | R.middletemporal | R.temporalpole | 2 |
|  |  |  |  |
| **BDI-II** | **ROI 1** | **ROI 2** | **T-stat** |
|  | L.inferiorparietal | L.middletemporal | 1.87 |
|  | L.medialorbitofrontal | L.precuneus | 2.38 |
|  | L.middletemporal | L.temporalpole | 1.94 |
|  | L.middletemporal | L.thalamus | 2.21 |
|  | L.precuneus | L.thalamus | 2.75 |
|  | L.middletemporal | L.caudate | 2 |
|  | L.precuneus | R.thalamus | 1.92 |
|  | L.hippocampus | R.thalamus | 2.44 |
|  | L.thalamus | R.inferiorparietal | 2.99 |
|  | R.caudate | R.inferiorparietal | 2.17 |
|  | R.thalamus | R.middletemporal | 2.27 |
|  | R.caudate | R.middletemporal | 3.35 |
|  | L.medialorbitofrontal | R.rostralanteriorcingulate | 1.87 |
|  | L.rostralanteriorcingulate | R.rostralanteriorcingulate | 2.34 |
|  | R.middletemporal | R.temporalpole | 1.74 |

**Table S11. Related to figure 6. Group differences (premanifest HD > controls)**:

**Resting state fMRI Track-On HD** (p = 0.036). ROI – regions of interest, T-stat – t-statistics.

| **ROI 1** | **ROI 2** | **T-stat** |
| --- | --- | --- |
| L.inferiorparietal | L.precuneus | 11.09 |
| L.isthmuscingulate | L.precuneus | 11.09 |
| L.inferiorparietal | R.inferiorparietal | 11.09 |
| L.precuneus | R.inferiorparietal | 11.09 |
| L.isthmuscingulate | R.isthmuscingulate | 11.09 |
| L.precuneus | R.isthmuscingulate | 11.09 |
| L.isthmuscingulate | R.precuneus | 11.09 |
| L.precuneus | R.precuneus | 11.09 |
| R.inferiorparietal | R.precuneus | 11.09 |
| R.isthmuscingulate | R.precuneus | 11.09 |

**Table S12. Related to figure 6. Group differences (premanifest HD < controls)**:

**Diffusion MRI Track-HD** (p = 0.018). ROI – regions of interest, T-stat – t-statistics.

| **ROI 1** | **ROI 2** | **T-stat** |
| --- | --- | --- |
| L.precuneus | L.temporalpole | 2.14 |
| L.precuneus | L.thalamus | 1.9 |
| L.entorhinal | L.caudate | 2.63 |
| L.isthmuscingulate | L.caudate | 1.97 |
| L.medialorbitofrontal | L.caudate | 1.81 |
| L.middletemporal | L.caudate | 4.86 |
| L.precuneus | L.caudate | 4.69 |
| L.frontalpole | L.caudate | 2.67 |
| L.temporalpole | L.caudate | 2.63 |
| L.thalamus | L.caudate | 3.63 |
| L.caudate | L.hippocampus | 3.55 |
| L.precuneus | R.caudate | 2.99 |
| R.thalamus | R.caudate | 2.36 |
| R.caudate | R.hippocampus | 2.6 |
| R.caudate | R.entorhinal | 3.72 |
| R.caudate | R.inferiorparietal | 3.6 |
| R.caudate | R.isthmuscingulate | 2.23 |
| L.frontalpole | R.medialorbitofrontal | 2.66 |
| R.caudate | R.middletemporal | 4.22 |
| R.caudate | R.parahippocampal | 2.54 |
| L.thalamus | R.precuneus | 2.46 |
| L.caudate | R.precuneus | 2.85 |
| R.caudate | R.precuneus | 2.74 |
| L.medialorbitofrontal | R.frontalpole | 2.07 |
| R.thalamus | R.temporalpole | 2.12 |
| R.caudate | R.temporalpole | 3.66 |

**Table S13. Related to figure 6. Group differences (premanifest HD < controls)**:

**Diffusion MRI Track-On HD** (p = 0.037). ROI – regions of interest, T-stat – t-statistics.

| **ROI 1** | **ROI 2** | **T-stat** |
| --- | --- | --- |
| L.frontalpole | L.caudate | 2.69 |
| L.medialorbitofrontal | L.hippocampus | 1.7 |
| L.medialorbitofrontal | R.thalamus | 2.57 |
| L.medialorbitofrontal | R.caudate | 2.32 |
| L.caudate | R.caudate | 2.18 |
| L.frontalpole | R.medialorbitofrontal | 1.76 |
| L.thalamus | R.medialorbitofrontal | 2.78 |
| R.caudate | R.middletemporal | 2.05 |
| R.thalamus | R.parahippocampal | 2.99 |
| R.caudate | R.parahippocampal | 2.74 |
| L.rostralanteriorcingulate | R.rostralanteriorcingulate | 2.11 |
| L.frontalpole | R.rostralanteriorcingulate | 2.52 |
| L.thalamus | R.rostralanteriorcingulate | 3.13 |
| L.caudate | R.rostralanteriorcingulate | 1.85 |

**Table S14. Related to figures 2, 4 and 6. Summary table for Track-On HD fMRI and Track-On HD and Track-HD diffusion structural cohorts showing depression and group NBS analyses.** Red – significant, Blue – approaching significance.

| **HADS-D** | **fMRI Track-On HD** | **Diffusion MRI Track-On HD** | **Diffusion MRI Track-HD** |
| --- | --- | --- | --- |
| **Premanifest (Binary)** | 0.008 | 0.036 | 0.917 |
| **Premanifest (Weighted)** | 0.118 | 0.363 | 0.0114 |
| **Controls (Binary)** | 0.935 | 0.931 | 0.925 |
| **Controls (Weighted)** | 0.933 | 1 | 0.795 |
| **Premanifest (Binary, drugs covaried)** | 0.024 | 0.0589 | 0.918 |
| **Premanifest (Weighted, drugs covaried)** | 0.075 | 0.223 | 0.075 |
| **Controls (Binary, drugs covaried)** | 0.95 | 0.44 | 0.485 |
| **Controls (Weighted, drugs covaried)** | 0.744 | 0.64 | 0.784 |
| **BDI-II** |  |  |  |
| **Premanifest (Binary)** | 0.026 | 0.019 | 0.255 |
| **Premanifest (Weighted)** | 0.157 | 0.592 | 0.045 |
| **Controls (Binary)** | 0.875 | 0.938 | 0.884 |
| **Controls (Weighted)** | 0.358 | 0.918 | 0.784 |
| **Premanifest (Binary, drugs covaried)** | 0.047 | 0.222 | 0.537 |
| **Premanifest (Weighted, drugs covaried)** | 0.14 | 0.221 | 0.012 |
| **Controls (Binary, drugs covaried)** | 1 | 0.931 | 0.887 |
| **Controls (Weighted, drugs covaried)** | 0.35 | 0.589 | 0.411 |
| **Group differences** |  |  |  |
| **Premanifest vs Controls (Binary)** | 0.036 | 0.0841 | 0.093 |
| **Premanifest vs Controls (Weighted)** | 0.657 | 0.035 | 0.0184 |
| **Premanifest vs Controls (Binary, drugs covaried)** | 0.133 | 0.0965 | 0.102 |
| **Premanifest vs Controls (Weighted, drugs covaried)** | 0.516 | 0.0304 | 0.0144 |

**Table S15. Reciprocal correlations (i.e. negative correlation with functional connectivity and positive correlation with structural connectivity).** **Depression NBS analyses for Track-On HD fMRI and diffusion structural cohorts:** Red – significant, Blue – approaching significance.

| **HADS-D** | **fMRI Track-On HD** | **Diffusion MRI Track-On HD** | **Diffusion MRI Track-HD** |
| --- | --- | --- | --- |
| **Premanifest (Binary)** | 0.999 | 1 | 1 |
| **Premanifest (Weighted)** | 0.883 | 0.977 | 0.454 |
| **Controls (Binary)** | 0.615 | 0.608 | 0.68 |
| **Controls (Weighted)** | 0.412 | 0.279 | 0.141 |
| **BDI-II** |  |  |  |
| **Premanifest (Binary)** | 0.998 | 0.117 | 0.611 |
| **Premanifest (Weighted)** | 0.874 | 0.57 | 0.561 |
| **Controls (Binary)** | 0.94 | 1 | 0.696 |
| **Controls (Weighted)** | 0.601 | 0.761 | 0.099 |

**Table S16. Related to figure 3. Apathy NBS analyses for Track-On HD fMRI and diffusion structural cohorts.** Red – significant, Blue – approaching significance.

| **Apathy** | **fMRI Track-On HD** | **Diffusion MRI Track-On HD** |
| --- | --- | --- |
| **Premanifest (Binary)** | 0.005 | 0.154 |
| **Premanifest (Weighted)** | 0.034 | 0.96 |

**Table S17. HADS-A NBS analyses for Track-On HD fMRI and diffusion cohorts.** Red – significant, Blue – approaching significance.

| **HADS-A** | **fMRI Track-On HD** | **Diffusion MRI Track-On HD** |
| --- | --- | --- |
| **Premanifest (Binary)** | 0.57 | 0.098 |
| **Premanifest (Weighted)** | 0.187 | 0.964 |

**Supplementary methods**

**Cohort**

The cohorts included participants from the London, Leiden, Paris and Vancouver sites of the Track-On HD study and the London, Leiden and Paris sites of the Track-HD study. From a total Track-On HD cohort of 243, participants were excluded due to; manifest disease 21, left handed or ambidextrous 24, 11 poor quality fMRI data and 1 subject lacking BDI/HADS data. This resulted in a Track-On HD fMRI cohort of 186 participants (92 premanifest HD and 94 controls) (see table S3). For the diffusion MRI cohort 35 poor quality DWI data sets were excluded, resulting in a diffusion MRI cohort of 151 (70 premanifest HD and 81 controls) (see table S4). The replication analysis was performed using a diffusion MRI Track-HD cohort, which we previously published (McColgan, et al., 2015), 1 subject was excluded due to lack of BDI/HADS data, resulting in 96 participants (50 premanifest HD and 46 controls) (see table S5).

**MRI Acquisition Track-On HD**

**Head coil information**: 3T MRI data were acquired on two different scanner systems (Philips Achieva at Leiden and Vancouver and Siemens TIM Trio at London and Paris), both using a 12-channel head coil.

**Structural T1:** T1-weighted image volumes were acquired using a 3D MPRAGE acquisition sequence with the following imaging parameters: TR = 2200ms (Siemens)/ 7.7ms (Philips), TE=2.2ms (S)/3.5ms (P), FA=10◦ (S)/8◦(P), FOV= 28cm (S)/ 24cm (P), matrix size 256x256 (S)/224x224 (P), 208 (S)/164 (P) sagittal slices to cover the entire brain with a slice thickness of 1.0 mm with no gap. In this protocol, a volumetric T2-weighted image (SPACE sequence in Siemens, VISTA in Philips) was acquired with the identical field of view, acquisition matrix, and slice thickness as the T1-weighted images, to provide complementary multispectral information for some methods of analysis. Scanning time was approximately 12 minutes.

**Diffusion:** Diffusion-weighted images were acquired with 42 unique gradient directions (*b* = 1000 sec/mm^2^). Eight images with no diffusion weighting (*b* = 0 sec/mm^2^) and one image with no diffusion weighting (*b* = 0 sec/mm^2^) were acquired from the Siemens and Philips scanners respectively. For the Siemens scanners, TE = 88ms and TR = 13s; for the Phillips scanners, TE = 56ms and TR = 11s. Voxel size for the Siemens scanners was 2 x 2 x 2 mm and for the Phillips scanners 1.96 x 1.96 x 2. 75 slices were collected for each diffusion-weighted and non-diffusion weighted volume. Scanning time was approximately 10 minutes.

**Resting state:** For resting state fMRI, 165 whole-brain volumes were acquired at a repetition time of 3s using a T2*-weighted echo planar imaging (EPI) sequence with the following parameters: TE 30ms, FOV 212mm, flip angle 80°, 48 slices in ascending order (slice thickness: 2.8mm, gap: 1.5mm, in plane resolution 3.3 x 3.3 x 3.3 mm^3^) and bandwidth of 1906 Hz/Px. Field maps were acquired with TR 1020 ms, TE1 10.0 ms, TE2 12.46 ms, FOV 212 mm and 2 mm slice thickness. All data were visually inspected by IXICO. Standardization of data acquisition across sites were performed based on previous suggestions (Glover et al., 2012). Scanning time was approximately 15 minutes. Scanning times of between 12-16 mins have previously been shown to increase the reliability of resting state fMRI connectivity estimates (Birn, et al., 2013).

**MRI Acquisition Track-HD**

**Head coil information**:  Siemens Tim Trio 3T (London/Paris) - 12 channel head matrix with HEA (head coil element anterior)/HEP (head coil element posterior) coils selected; Philips Achieva 3T (Leiden) - 8 channel SENSE (SENSitivity Encoding) head coil.

**Structural T1:** T1-weighted image volumes were acquired using a 3D MPRAGE acquisition sequence on 3.0 T Siemens (London and Paris) and Phillips (Leiden) whole body imager with the following imaging parameters: TR = 2200ms (Siemens); 7.7ms (Philips); TE=2.2ms (Seimens); 3.5ms (Philips); flip angle =10◦ (Seimens); 8◦ (Philips); FOV= 28cm (Seimens); 24cm (Philips); matrix size 256x256 (Seimens); 224x224 (Philips), 208 (Seimens); 164 (Philips) sagittal slices to cover the entire brain with a slice thickness of 1.0 mm with no gap. Scanning time was approximately 10 minutes for T1-weighted and 9 minutes for diffusion weighted acquisitions.

**Diffusion**: Diffusion-weighted images with 42 unique gradient directions (*b*=1000 sec/mm^2^) were collected with either seven images (Siemens) with no diffusion weighting or one image with no diffusion weighting (Phillips). For scans collected in London, dimensions were 128 pixels × 96 pixels × 65 slices per volume, with TE=84ms and TR=7600ms; for Paris, dimensions were 128 pixels × 128 pixels × 75 slices per volume, with TE=88ms and TR=13100ms and for Leiden, dimensions were 112 pixels × 112 pixels × 55 slices per volume, with TE=56ms and TR=8078ms. Voxel size for Siemens scans was 2x2x2mm^3^ and for Phillips 1.96 x 1.96 x 2mm^3^. Scanning time was approximately 9 minutes.

**Functional MRI preprocessing**

Regression of noise ROIs (without global signal regression), band-pass filtering and the calculation of bivariate correlations were performed as this processing strategy has been shown to produce the most reliable graph theory measures (Andellini, et al., 2015; Aurich, et al., 2015; Liang, et al., 2012). For each subject the T1 image was segmented into grey matter, white matter and CSF. WM and CSF masks were eroded by one voxel to avoid partial volume effects with grey matter. The first 5 principle components of signals from the eroded white matter and CSF masks were regressed out, using the anatomical Compcorr method (Behzadi, et al., 2007), along with 6 movement parameters. The BOLD time series was then band pass filtered between 0.009-0.08 Hz. Band-pass filtering was performed after regression in order to avoid the re-introduction of noise (Hallquist, et al., 2013). Bivariate-correlation coefficients were calculated by extracting the residual BOLD time series for each ROI or seed pair across the 76 brain regions (resulting in 2,850 possible functional connections) and applying a Fisher transform in order to normalize the data.

**Cortical modules and depression**

The Louvain method for community detection calculates brain modules by splitting the brain into non over-lapping groups of regions, which maximizes the number of within-group connections, and minimizes the number of between-group connections. A resolution parameter is chosen in order to detect either small modules (<1) or large modules (>1). In this study a value of 0.5 was chosen in order to identify large modules and thus avoid excluding brain regions that may be implicated in depression. This resulted in decomposition of the brain into 2 modules.

**Network based statistics**

Using this method, a test statistic was calculated for each connection independently. A primary threshold (p < 0.05, uncorrected) was then applied to form a set of supratheshold connections. Permutation testing is then used to ascribe a p-value controlled for FWE to each set of suprathreshold connections. For each permutation the test statistic is recalculated, after which the same threshold is applied to define a set of suprathreshold connections. The maximal component size for each permutation is determined giving a null distribution of maximal component size. Finally the FWE corrected p-value of the observed component size k is estimated by finding the proportion of permutations for which the maximal component is greater than k. The FWE adjusted p- value is set at 0.05.

Based on observations from the literature that depression shows positive correlation with functional connectivity and negative correlation with structural connectivity in the DMN and basal ganglia (Gong and He, 2015), positive correlations were tested in the resting state fMRI analysis, while negative correlations were tested in the diffusion MRI analysis (thus a one-tailed correlation for each analysis).

**References**

Andellini, M., Cannata, V., Gazzellini, S., Bernardi, B., Napolitano, A. (2015) Test-Retest reliability of graph metrics of resting state MRI functional brain networks: a review. Journal of neuroscience methods.

Aurich, N.K., Alves Filho, J.O., Marques da Silva, A.M., Franco, A.R. (2015) Evaluating the reliability of different preprocessing steps to estimate graph theoretical measures in resting state fMRI data. Frontiers in neuroscience, 9:48.

Behzadi, Y., Restom, K., Liau, J., Liu, T.T. (2007) A component based noise correction method (CompCor) for BOLD and perfusion based fMRI. NeuroImage, 37:90-101.

Birn, R.M., Molloy, E.K., Patriat, R., Parker, T., Meier, T.B., Kirk, G.R., Nair, V.A., Meyerand, M.E., Prabhakaran, V. (2013) The effect of scan length on the reliability of resting-state fMRI connectivity estimates. NeuroImage, 83:550-8.

Gong, Q., He, Y. (2015) Depression, neuroimaging and connectomics: a selective overview. Biological psychiatry, 77:223-35.

Hallquist, M.N., Hwang, K., Luna, B. (2013) The nuisance of nuisance regression: spectral misspecification in a common approach to resting-state fMRI preprocessing reintroduces noise and obscures functional connectivity. NeuroImage, 82:208-25.

Liang, X., Wang, J., Yan, C., Shu, N., Xu, K., Gong, G., He, Y. (2012) Effects of different correlation metrics and preprocessing factors on small-world brain functional networks: a resting-state functional MRI study. PloS one, 7:e32766.

McColgan, P., Seunarine, K.K., Razi, A., Cole, J.H., Gregory, S., Durr, A., Roos, R.A., Stout, J.C., Landwehrmeyer, B., Scahill, R.I., Clark, C.A., Rees, G., Tabrizi, S.J., Track, H.D.I. (2015) Selective vulnerability of Rich Club brain regions is an organizational principle of structural connectivity loss in Huntington's disease. Brain : a journal of neurology.

Penney, J.B., Jr., Vonsattel, J.P., MacDonald, M.E., Gusella, J.F., Myers, R.H. (1997) CAG repeat number governs the development rate of pathology in Huntington's disease. Annals of neurology, 41:689-92.
